# Miscellaneous

**Published:** 1857-12

**Authors:** 


### Miscellaneous.

### DENTAL SOCIETIES.

We sat down, under a sense of duty, to write a short article on the advantages to be derived from dental associations. We *felt* full of the subject, but our feelings were slow in assuming the form of thoughts; and even the thoughts refused to be represented by words. In short, we were not in the mood, and, therefore, could not write. But "there is a Divinity that shapes our ends," for here is an article in the editorial of the "Western Lancet," signed "R," (who's R. ?) which says, of medical societies, nearly all we aimed at, and something besides, in regard to dental associations. Now, reader, just change the word " Medical " to *dental*, and peruse the article.

Medical Societies.—The importance of local medical societies as a means of improving and elevating our profession, does not appear to be generally appreciated. Evidence of the want of interest there is upon this subject, is to be found in the report of the committee on medical societies presented to the State society last year. Ohio contains about eighty counties, yet only twenty answers were received to the circular of the committee making inquiries in regard to local societies. There seems to be only about half a dozen societies in the State in active existence; of the others, some show faint symptoms of vitality at long intervals, others are in a state of suspended animation, and many more are dead and beyond any power of resurrection.

That much positive evil results to the profession from this state of listless inactivity, and that more positive advantage is lost, we think will not be denied by any one who has given a moment's consideration to the subject. The " elevation of the profession " seems to be a favorite topic with its writers and speakers, to judge from the frequency with which it is chosen as a subject for lectures, addresses, and dissertations; and we are as fully aware as any one that the profession does not occupy as high ground as it should. Now we believe that there are no truths more evident than that the elevation of any body of men must proceed from within itself, and that no false position can be forced upon it by external circumstances alone. Our profession can not be honorable and respectable unless its individual members are educated to such a degree, and act in such a manner as to command the respect of the public, and there is no more powerful means of watching and controlling individual conduct than that afforded by a local society. Individuals are the *cells* in whose proper or improper action depends the health of the whole,—united into societies, they become the *tissues* of the body medical, which possess the power of regenerating those cells likely to decay, and casting off those no longer necessary or beneficial to the general economy.

No more striking instance can be found of the power of united and persevering action of the members of a profession in ameliorating its condition, than is presented by the teachers of this State, and we point to them as an example to be followed, although we regret the necessity which compels us to go beyond our own ranks for the best instance of advance and self-improvement. Within our own remembrance, teaching was certainly not a remunerative calling, and could scarcely be termed an honorable one—it was pursued only temporarily by those on their way to something better—wages were miserably low, qualificatious not strictly inquired into, so that many were engaged in it who were not fit for their calling, and it was far too unattractive to tempt any one to devote a life to it. Now things are entirely changed; every city contains many men who make teaching their profession, they occupy an honorable position in society, and receive good salaries for their services. And throughout the country the average wages of teachers have been doubled within the period of which we speak. If we ask how these changes have been effected, we shall find it has been by the united action of the teachers themselves, having the object directly in view; they have held conventions and institutes, formed societies, increased their own qualifications, and insisted on increased qualifications from those seeking admission to their ranks, and by these and other means have educated the public mind to estimate at their true value the services rendered by them.

Now if our profession does not occupy the position, and our services do not receive the reward which they should do, it is plain that we are in the position formerly occupied by the teachers, and it is but fair to infer that the same means which benefitted them will benefit us. There is this much in common to both—the *faithful* discharge of the duties of each requires something more than pecuniary gain. Without being deeply impressed with the importance of his mission, and without a sole reference to conscience as his judge, no man can be a good teacher or a good physician. Without these qualifications, he may belong to an honorable profession, but he is not an honorable member of that profession.

Were the facts above alluded to not before us, we should think it superfluous to mention the advantages which result from the foundation of medical societies. But as so few of these associations are in existence, we are forced to the conclusion that it is because the benefits they confer even upon the members themselves, are not generally recognized. We therefore call attention, very briefly, to a few of the most prominent.

1. *They add very much to the recorded experience of the profession.* The science of medicine has been often likened to an edifice, of which the facts observed by individuals are the stones, and the simile is a good one; but thousands of these stones which would extend or ornament the noble structure, are lost every year for want of medical societies. They record, select and publish those facts coming under the observation of individuals, often of so much interest and of so much value, which would otherwise never be preserved, and the elder members of the profession have stores of valuable experience, which in societies would be brought out for the benefit of their younger brethren or of the fraternity at large.

2. *Societies call out and encourage talent.* This is especailly the case with young men who have just commenced their professional life. Without any stimulus to develope, or any field in which to exercise their powers, they settle down into a quiet routine life, careless of their own progress, and of the progress of the profession; with a society to bring them into contact, and honorable rivalry with their professional brethren, they may become active, studious, and *living* members of the profession. Sir Charles Napier, the soldier, said: " Man! Man! thou art a beast, in whose sides the spur should be ever plunged!"

3. *They powerfully conduce to individual improvement.* First, by encouraging the study of those important and fundamental branches, physiology and pathology. But very few men will speak upon a subject before their equals, without being familiar with it in all its bearings, and in the discussions which spring up in medical societies, a reference to first principles is constantly necessary. A young man soon finds, therefore, that he needs a better acquaintance with principles and with authorities, to enable him to hold his own against active and intelligent adversaries. Second, by encouraging a habit of close observation. Every one who tries to add his share to the proceedings of the association, will watch his cases far more closely, and record them more carefully, when he intends to bring them before the notice of his medical brethren. If he has no object of this kind in view, they are too apt to pass entirely unrecorded and be forgotten.

4. *They promote harmony in the profession.* More than half the doctors' quarrels which so frequently occur, and which so deeply disgrace us, originate with indiscreet friends of the parties. They magnify, distort and misrepresent facts, until the most trivial circumstances assume gigantic proportions. Where there is a medical society, there is frequent opportunity of settling these misunderstandings; falsehoods can be exposed and misrepresentations cleared up; by intercourse with our medical brethren, many prejudices are removed, we find many good points in those we thought before altogether bad, and even sympathies will spring up, as we find them laboring as devotedly as ourselves for the common good.

5. *Societies strengthen the profession against its enemies, external and internal.* However much quackery may differ in the color of the garment with which it clothes itself, or in the note with which it pursues its prey, it never treats the regular profession in any other way than by open hostility. All schools, all tribes, all doctrines, unite upon this point. Without union and harmony, the profession can do but little against this hydra. With them it can do much. The formation of a society will always do this much, and it is no little—it will separate the wheat from the tares. The public mind needs to be educated upon many points in regard to our profession; upon none more than upon the difference between the regular and scientific physician, and the dishonest or ignorant quack.

We are well aware that the worst enemies of the profession are not those who stand outside of its ranks. Those who are connected with it in appearance, yet are its enemies in fact, are more dangerous to its welfare, and more difficult to overcome. They are traitors to the cause they have enlisted in, and like all traitors, can do more injury than the foe. We are sorry there are such men, and still more sorry that they are not foes. An instance is given in the report already alluded to, where one man of this character was the means of breaking up the local society entirely. There are two characteristics by which this class may generally be known: they will shake hands with the quackery for the paltry consultation fee which drops into their pocket during the dirty process, and they are opposed to the medical society of the county or district where they live. Against such men as these there is no resource without an organization. The other physicians are too often at their mercy, and the profession is degraded to the level of those with whom it is thus forced to associate. Wherever such men are found, the other members of the profession should unite and act faithfully and cordially for their mutual good—compel the obnoxious individual to join the organization under the Code of Ethics of the American Medical Association, or to take his place in the ranks of quackery.

"Under which king, Bezonian? speak or die,"

should be forced upon them, until they are compelled to answer. If a firm, consistent, and dignified course is pursued by the society towards these individuals, we believe it will always result in victory to the association, and never to the individual.

6. *By means of Societies the remuneration for our professional services can be increased.* We place this last, where it should be placed, as a *motive.* yet it is a subject of no small importance to every one of us. In no other profession are men so poorly paid in proportion to the physical labor and essential anxiety expended in its prosecution. In no other calling is the number of those who acquire a competence, so few as in the medical profession. While the expenses of living have increased many fold, the medical fee bill, in many sections at least, remains the same as it was ten or twenty years ago. There is no remedy for this but united action on the part of the profession, and although some may deem it for their interest to continue doing business on the "cheap John" principle, the profession will be better off without those who can descend so low as to recommend themselves to the public on the score of being *cheap.*

There are many more advantages which might be urged in favor of societies. We know of no objection to them. There are some difficulties in the way everywhere, but none, we believe, that can not be overcome, if met in the proper spirit. To some of these we intended to allude, but our article is already too long, and we must defer this part of the subject to another number. We have brought the matter forward now, because a season of the year is approaching, favorable to the formation of associations among the profession, and we feel that we can not urge too strongly its attention to the vital importance of forming societies in every part of country where they do not already exist, and of rekindling fires upon altars where it has ceased to burn. R.

### METHOD OF PROMPTLY RELIEVING FACIAL AND DENTAL NEURALGIAS.

This method consists in turning into the meatus auditorius from four to ten drops (according to the age and sensibility of the patient) of the following fluid: then to close the opening of the ear by means of a little cotton, and to cause the patient to hold the head inclined for some minutes to the side opposite to the seat of the pain, so that the liquid may remain in the bottom of the ear. This preparation is thus made: ℞ Take of the extracts of opium, of belladona and of stramonium each *one part;* of distilled cherry laurel water *twelve parts.* Dissolve and filter.

Although this preparation may be only extemporaneous, it may nevertheless be preserved, if care is taken to keep it cool, and to pour on its surface from two to four drops of sweet almond oil.

It is very rare that with the use of this liquid, relief is not obtained in a few minutes; indeed, the patient is almost always asleep in half an hour, whatever may have been the severity of the pains, and that without having been in the least danger.

Absorption takes place almost as rapidly as from a denuded surface, and it is therefore unnecessary to blister the patient when we wish to use narcotics, since they act almost as rapidly by the auditory passage.

If it should happen that, at the end of eight or ten minutes, the pain does not yield to the remedy (which sometimes happens when the quantity used has been too small, or when we have to treat a neuralgia which has already required the use of narcotics in any way,) it is necessary then to use a second dose, at least equal to the first, but in the opposite ear, in order to obtain promptly that relief which is only too frequently momentary in facial neuralgias of long standing.

The preference which I give to this aqueous solution over those which contain alcohol, such as laudanum and other narcotic tinctures, arises from having used both upon myself for several years for a facial neuralgia, and observing that the latter produce a sensation of quite acute pain at the moment of their use, and not being always as successful as the former, which causes neither heat nor smarting, and is more certain in its effects.—*M. Andre, in the Revue de Therapeut.*

### ON GELATINIZED CHLOROFORM.

Translated from the Revue de Therapeutique, by the Editor.

My Dear Sir: I think it time to reply to your note on Gelatinized Chloroform; although the details on this subject are not abundant, I send them, such as they are. According to the experiments of M. Aldiz, y Fernandez, the gelatinization in cold of chloroform takes place in equal proportions of albumen (white of egg) and of chloroform. The mixture being shaken, assumes the consistence of collodion, and after three hours rest it acquires a gelatinous form. Pure chloroform, which should be preferred, affords a whiter and more consistent preparation than that obtained from the chloroform of commerce.

When obtained by means of the salt baths, the proportions are not the same. One part of albumen to four parts of pure chloroform are mixed in a flask, which is immersed in a bath of fifty to sixty degrees; this process requires but four minutes.

I have employed the preparation in several cases, in all of which I have prescribed the article made in the cold. If I say nothing of the second process, it is because I have found the first more simple and successful. The following are the results of my observations:—Following violent exercise in a play called the mutton leap (saute-mouton), the son of Alasauniere, Veterinarian of a haras complained of acute pain at the point of the right scapula. This pain, so severe as to induce great anxiety in the patient, occurred suddenly two or three times in the twenty-four hours, without apparent cause, both during rest and exercise of the body. In the intervals of the pain, our young patient could not point out with his finger its exact locality. All the movements of the trunk and shoulder were free, and painless. After two or three applications of the gelatinized chloroform the pain disappeared entirely.

A lady on a visit to M. Alasauniere complained of severe pain in the elbow, which nothing relieving for several days, she was informed of the recent success of the gelatinized chloroform—a single application cured her.

Widow Villebois, following prolonged mental sorrows, was attacked with right facial neuralgia; two applications of the remedy were sufficient; 8 days afterwards the neuralgia reappeared in an intermittent form; here the anesthetic failed, and I had recourse to sulphate of quinine, which was successful.

M. Gouin, employed in the police, convalescent of a pleurisy, sat for a quarter of an hour in a damp basement room. He suffered the following night from rheumatic pains in the muscles of the left thigh—three applications of the gelatinized chloroform cured him.

Anna ———, attacked with chronic metritis, experienced an exacerbation of the hypogastric pains, which resisted the influence of belladonna, was treated by the application of the remedy to the neck of the uterus on a tampon of cotton, and by friction over the abdomen; two applications of the remedy relieved her.

In Spain, M. Rodriguez publishes, in the *Eco de Los Cirujanos*, some new facts in favor of the efficacy of gelatinized chloroform, topically applied. The first case was one of long standing gout; where the intolerable pains were located in the joints of the feet, of the hands, and the inferior portion of the abdomen; on the influence of general evacuations and warm baths the pains were ameliorated but not cured. Relief from the first application of the solidified chloroform, and total cure of pain and swelling by the fourth.

Case 2.—Morante, 42 years old, subject to lumbago, was attacked in June 1856. Antiphlogistics, warm baths, emolient cataplasms, and anodynes without success; cured by three applications.

Case 3.—Success of the remedy in rheumatic pains of the muscles of the chest and of the scapular region.

Case 4.—Lumbar pains produced by a fall, and treated by Peau sedative without effect; cured in a few days by local applications of the remedy.

I have often observed, that when instead of direct friction the remedy is held for an instant in contact with the skin in the open air, or a strong dose is applied by friction, the surface becomes superficially cauterized, accompanied with severe pain. (We have observed the same result from the local application of chloroform itself, and distinctly remember a case of super-orbital neuralgia, in which the anesthetic was applied by means of a piece of cotton to the brow at the exit of the nerve, vesication was almost instantaneous, with intense local pain for an instant, and entire relief of the neuralgia for several days.—Ed.) Nothing of the kind follows the application of liquid chloroform, when its volatilization is not prevented, by some impermeable body or tissue. This little accident is an excellent proof of the topical value of the preparation of M. Aldiz y Fernandez. It retains the chloroform in contact with the living tissues, long enough to develop its anesthetic property. The superficial cauterization of the skin had no therapeutic value, and need not be inflicted. Friction with gelatinized chloroform, leaves under the fingers a quantity of inert albumen, which should be removed by the hand; this little inconvenience is of no consequence in the application of the remedy.

I can not terminate this article without expressing my regret that gelatinized chloroform is not used in France; and again inviting practitioners to try this excellent remedy, which is applicable in all pathological conditions.

DR. MASSART.

How will it operate as an application to sensative dentine and exposed nerves?

It is represented as a valuable application in the treatment of neuralgia.

We hope the profession will try it and report. ED.

*Gelatinized Chloroform.*—Dr. Massart gives, in the *Revue de Therapeutique Medico-Chirurgicale*, the following methods of making a solid preparation of chloroform:—1st, mix equal parts of white of egg and chloroform; shake the mixture, and let it stand for three hours. Or, 2d, take one part of white of egg and four parts of chloroform; put them in a bottle and plunge it completely in a water bath of the temperature of from 120 to 140 degrees Fahr.; gelatinization takes place in four minutes. This preparation is to be rubbed on a painful part, and, according to M. Massart, its effects in relieving pain are remarkable. He prefers the cold method of making it. If allowed to remain long in contact with the skin, it produces a superficial cauterization and pain.

---

*Syrup of Borax.*—In cases of laryngeal catarrh, M. Trousseau prefers this syrup to the use of gargles. His formula is four drachms of borax to ten ounces of simple syrup. A teaspoonful may be taken seven, eight or ten times a day, care being taken not to drink immediately afterward; that the contact of the salt with the affected mucous membrane may be prolonged as much as possible.—*L' Union Medicale*

### VALERIANATE OF AMMONIA,

### AS A REMEDY FOR NEURALGIA.

It will be recollected that in the first number of our present volume we publish an article, from the Montreal Medical Chronicle, reporting very remarkable success, by Dr. Declat, in the treatment of Neuralgia with the above-named medicine. We have seen as yet no report of cases in our American exchanges—but have received many private letters asking how the article should be administerd, and what is the dose? Having had recently under our treatment two obstinate cases of temporal and facial neuralgia, and having failed to afford relief by any of the ordinary means—revulsives, tonics, quinine, and even opium failing to abate the pain—we referred to the report of Dr. Declat with the view of resorting to this new remedy. It was now that we were able to appreciate the embarrassment of our correspondents about the *dose.* The cases in Dr. D.'s paper are carefully reported, apparently, but the dose certainly veiy indefinitely stated.

" A teaspoonful taken in the evening modified the pain at night and rendered it bearable. Two teaspoonfuls next day gave complete relief."

Now this " teaspoonful " we *suppose* was a solution, but of what strength? Was the original preparation used by Dr. Declat in fluid form, or was it, as we now receive it from the chemists, in the form of a *salt.* No definite dose is given, and hence the embarrassment in its administration. Wishing to try the efficacy of the article, we made a solution at first of 10 grains to the ounce of water. Of this, we directed our patient to take one teaspoonful three times a day, till the two ounce solution was exhausted. At the end of the second day, he increased the dose to one and a half teaspoonful, and reported a slight amelioration in his sufferings.

On preparing for this patient a second vial, we dissolved 32 grains of the salt of valerianate of ammonia in two ounces ef water (two grains to the drachm), directing the dose as before, viz. 1½ teaspoonful three times a day. When this was exhausted, we prepared a solution which contained three grains to each drachm of water, still advising the above doses.

*Case* 2d.—This was a negro woman, who had suffered from severe neuralgic pains in the temporal and occipital regions for six weeks. Quinine and other remedies failing, we administered the valerianate of ammonia in doses of four grains to the teaspoonful of water, three times a day. On the second day the pains were much abated, and under the continued administration of the remedy, in similar doses, the distressing symptom has disappeared.

Valerianate of ammonia, as we have seen it, presents the characters of a dirty-looking deliquescent salt, emitting a strong odor of valerian, and we may add, for the information of our readers, *costing* four dollars an ounce.

In relation to our success with this remedy in the above cases, we have to report that it has been very satisfactory, but at the same time we must say we would have been greatly disappointed did we not measure our credulity in this remedy by the good, old, *safe* rule "of believing just about one-half of what we hear" in relation to the effect of remedies.

When we read Dr. Declat's article, the case of "Madame the Marchioness of Fontanelle, who had been attacked six years ago with facial neuralgia of the most severe description," and had passed through the hands of Legrand, Jobert (de Lambelle), Sedillot and Velpeau, besides a residence at mineral springs, and the best alterative and tonic treatment, unrelieved, and "when the agony was unendurable and the patient in despair," relief came suddenly from three teaspoonfuls of a new remedy, we must confess that we measured our belief rather by what would *satisfy* us than by what we saw written in the report. If the relief was *half* as prompt in all cases, even with double the amount of medicine, we would be satisfied.

In making the above remarks, we would have it fully understood that we do not wish to doubt, in the least, the *correctness* of the *statement* of the reporter; far from that—we give full credence to the report, so far as relates to the two cases mentioned there; but two cases were too small a number to judge of the *general* efficacy of a specific remedy; the effect might be attributable to accident or coincidence, a *post hoc* merely and not a *propter hoc*; so, in the end, we were not disappointed much when our patient was only *partially relieved* after taking the remedy for about *ten days*. Our conclusion in relation to the remedy, after this partial trial, is, that it is a very useful article in the treatment of this form of neuralgia, so far as can be determined by the observation of these two cases and the testimony of the original favorable report of it: and we can further say, to those who wish to administer it, that our own careful experimental administration of it, gradually increasing the dose, proves that in doses of *four grains of the salt* to the *teaspoonful of water*, the remedy has no *injurious* effect upon the system, but its effects have been highly satisfactory. Whether or not we have yet reached the *full* dose used by Dr. Declat, we can not say; that must be determined either by farther experiment or by a more definite statement from that distinguished gentleman himself.—*Editorial—from Southern Med. Jour.*

---

NITRATE OF SILVER IN TOOTHACHE.—The common methods of destroying the nerve in a tooth without extracting it, are the application of nitric or sulphuric acid, or a red-hot wire, but these are very painful expedients.

Some time ago I tried the application of a small piece, about the size of a pin-head, of stick caustic (nitrate of silver) in the hollow of a decayed tooth, from which I was suffering extreme pain. To my great surprise the pain instantly ceased on the caustic touching the nerve, and I had not a return of the toothache for twenty-four hours; when I again applied the caustic, and again got immediate relief, which continued other twenty-four hours. I had occasion to make a third application of the caustic, but since that time, now some months ago, I have not been troubled with the toothache.

This cure will be found ineffectual if gum-boils accompany the toothache, for in that case the decayed part of the tooth is on the outside of it, and therefore the application of the caustic to the interior of the tooth can do no good.—DR. JAMES JOHNSTONE, in *London Lancet*

A solution of Nitrate of Silver has long been employed as an application to sensative dentine, and in many cases with perfect success.

It has also been used to some extent in the treatment of exposed nerves, both for restoring to health when sleightly diseased, and for destroying them when that result was desired. For the latter purpose a more concentrated solution, and sometimes the crystal was used. But we were not aware that it is a specific for toothache.

One case is not quite sufficient to establish its efficacy for that purpose.

The reason given why it is ineffectual when gum-boils accompany the tooth-ache, is original. *Cause why*, "the decayed part of the tooth is on the outside of it."

Pine apples grow on pine trees, for, if they don't where do they grow—say? Try again DR. JOHNSTON.

### EXTRACTING TEETH.

We have received a communication from Dr. Curlee, of Florida, asking us to say something upon the subject of extracting teeth, as he regards it as a neglected subject. He believes there is a great deal of *injudicious extraction*, as well as *premature.*

As regards premature extractions, we have said more perhaps than any other writer, and what we have said, has been fearless and candid, although it was in conflict with some of the most popular living authors. Under the treatment of irregularities, we have been giving what we regarded as proper directions for extracting deciduous teeth, and in as plain a manner as we knew how, and we did it as our bounden duty.

The *order* of eruption of the permanent teeth, is the order of extraction for the first set, and not in *rotation* as has been taught and practised, removing laterals, then canines, and then first molars, but first molars after the incisors, then the second as a general rule, and lastly canines; I mean the first set. We do not write to censure, but to correct the wide spread mischief which has resulted from an erroneous view which was first conceived of—a proper or correct system of extracting the deciduous teeth. We have also endeavored to show that it is not a correct practice to extract a deciduous tooth, even though the second is coming out of line, unless there is room for it in the arch by removing one deciduous tooth only; but let the first remain until there is sufficient space developed for the second, as there is more hope of a growth of the arch with both teeth in, than when one is extracted. We think we have sufficiently illustrated that by cases already published. We never remove two deciduous teeth for one permanent one, no matter what apparent irregularity may exist, if by letting the shedding be performed by nature, and we should find at the age of fourteen or sixteen years of age, that there has not been sufficient expansion of the jaw to allow all the teeth to fall in line, by having all the advantage of the first set, then we find it time enough to extract one or more of the permanent teeth as the case may be. We do not extract a deciduous tooth at the age of four, five or six years, merely because it is an occasional cause of pain. We treat toothache in children as in adults, to preserve the mouth from contracting. If, in this degenerate age, nature is destroying the deciduous teeth prematurely, by decay, and only making a temporary destruction, it is no reason why we should aid her in inflicting a permanent injury for life. It is not aiding nature to extract a tooth because it aches; it is true, it causes us a great deal of trouble to aid nature in retaining the first teeth in the mouth, long enough to fulfill the purposes of their development. If it be a burden to us to labor hard, and even expose a little sufferer to temporary pain for a time, it is no reason why we should, by extreme measures, rid ourselves of it, by destroying the mouth of an innocent creature for life. It is our duty, as much as in our power lies, to palliate pain in affliction as much as possible, for a more permanent good; it is mistaken sympathy to get rid of a temporary evil by inflicting a permanent injury.

As regards the injudicious extraction of teeth, there is little hope of ever improving that evil to a very great extent. As long as patients work themselves up by a desperate effort to the "sticking point," they will be liable to induce dentists to act rashly in regard to the number of teeth extracted at a sitting, and the condition of the health of the patient at the time. We do not regard the extraction of a tooth of a female during pregnancy as injudicious, although it might have been highly so in the case referred to by Dr. Curlee. Many female patients are more liable to toothache, during and especially near "full term," than any other time, as there is an increased exhilaration of the circulation, and increased sensibility of the nervous system and determination of blood to the head, all of which circumstances favor toothache, on the same principle that the increased or febrile exacerbation of the evening, or forepart of the night of any one in health, accounts for the fact that patients complain more of toothache at that time than any other in the twenty-four hours; if a patient comes to us in the morning, and says that they have been suffering all the forepart of the night with the toothache, but are not suffering at the time they call, we at once infer that the pain had been induced by an exposed nerve. There is an increased sensibility of the pulp by exposure, as well as a slight congestion of the part, and during excitement or febrile exacerbation, pain will be induced. If, in a female patient, there is no superstition as regards the operation during pregnancy, and the extraction of the tooth does not involve much time, extraction would do less harm than to allow the patient to be exposed to prolonged suffering during such a critical period.

We are not a "tooth-puller" in any sense of the word; we do not extract a tooth for any patient, unless there is a dire necessity for it. Some patients wish a tooth extracted because it is unpleasant, or looks badly, or induces swelling of the gums sometimes, or may cause other teeth to decay, or make the breath offensive, or some other of an hundred reasons. Teeth are not always in a condition to extract; sometimes they are too much inflamed when the patient calls on us, such as in the case of the formation of abscess. It is never necessary to extract a tooth for pain in case of an exposed nerve, as pain from that cause can always be stopped. The tooth may be too much decayed, the crown may be decayed to the gum, and the alveolar process can not well be cut away, to get hold with an instrument sufficiently to extract it, and it may become the cause of a great deal of suffering on the part of the patient, and labor on the part of the dentist. Hence, it would be the least of two evils to let it alone, until the gum shall have become softened, and the alveolar process absorbed. We are in the habit of waiting until a condemned tooth gets in condition for extracting, except in some extraordinary cases, and in such cases we can apply no rule, for they must be governed by circumstances existing and decided upon by the patient and the dentist at the time, and which depends upon the exercise of a judgment, which time, reflection and education only can develop. No dentist ought to extract a tooth for a female patient during pregnancy, without the knowledge of her medical adviser, although dentists who possess a good medical education, are often consulted by physicians, as regards the propriety of extracting teeth in patients in various conditions of health.

Some dentists contend that every tooth that can not be plugged, ought to be extracted, no matter what age the patient is at the time they may be getting their mouth "put in order;" this is not true, except to a dentist who is blind or reckless to the changes it will produce on the mouth of a young patient, say at any period under twenty years of age. We are now treating four lamentable cases of irregularity, caused by *injudicious* extraction of *permanent* teeth; one patient is sixteen years of age, one eighteen, one twenty, and one twenty-four. Permanent teeth are necessary in the jaws, until they are properly developed, and until the teeth have become permanently articulated, so that when a tooth is extracted, it will not cause a derangement of that important condition.

The parts change more by extracting a tooth for a patient under twenty than when older; hence, when a tooth is extracted for a young patient, the parts will close up, not "fill up," by contracting the jaw, and produce defective articulation with its fellow, which may end in a serious evil. We will draw especial attention to this in our next article on irregularities.—*News Letter.* J. D. W.

---

### "ADVICE TO THOSE WHO USE AMALGAM."

Never fill a tooth with amalgam, or any other "succedaneum" which you can, by the exercise of skill and care, fill with gold.

If you determine that the crown of a tooth (the nerve or nerves of which are exposed) is too frail to bear filling with gold in a solid and proper manner, destroy the nerves with arsenious acid, and having thoroughly removed them, fill the fangs carefully with gold. Upon this you may build a crown of amalgam, with a reasonable hope of its being serviceable.

In all teeth liable to be attacked by periosteal inflammation, the proper plan will be, to treat the fangs and fill them with gold, prior to any attempt to fill the crown with anything. When the fangs are well filled, and a few days have elapsed without inflammation, the crown cavity may be filled as safely with amalgam as with gold, so far as periostitis is concerned.

The use of amalgam in the practice of a skillful and dexterous manipulator will be very limited; still, there are cases which occur in every man's practice, where he can not use gold or any material which requires force to make it pack, and yet it may be very desirable to save the tooth for purposes of mastication. In such cases I deem it the duty of every honest dentist to use such material as he thinks will make the organ useful for the longest period.

If the operator decides upon using amalgam, and thinks his patient might object to it, from the odium which has heretofore been heaped upon it, it is then his plain duty to explain to him the nature of the material, the objections which have been made to it, with his views of the worth of those objections, and then, let the decision for it or against it rest with the patient, either to fill it with amalgam or extract it.

Never allow any one to say he was deceived by the use of an article in his teeth, which, if he had known the nature of, should not have been put there.

In using amalgam as a filling for any cavity, always remember, that to expect success, you must take the same amount and kind of care in forming your cavity, and the same care in keeping it dry while packing it, as in a filling of gold—and also, that the more friction you can give the surfaces with the burnisher, the more compact and well will your filling harden, and bear a higher polish when hard.

I wish it to be fully understood, that I advocate amalgam fillings in the practice of those who can receive liberal compensation for time and skill, only in such cases as would otherwise have to be extracted. I have seen hundreds of mouths in which amalgam fillings have been placed, some recently, and others several years since, and have never, in any case, seen any injurious systemic effect.

There may be, and no doubt there are, thousands of teeth extracted and lost, in the practice of those who are obliged to operate for very low fees, (as in country towns and villages,) which might be rendered serviceable for many years, by the use of a cheap material.

Dental services are now demanded by all classes of people, and there are comparatively few who can afford to pay from 30 to 50 dollars for a fancy operation, such as building up of a gold crown, and for such cases provision ought to be made.
—*News Letter.* ELISHA TOWNSEND.

The writer of the above seems to be now much nearer a correct position on this subject than when he wrote an article upon it two years ago.

The advice contained in the first sentence is very good, and if all should come to that point the use of amalgam would be beautifully narrowed down.

It is here interdicted in all cases where by the "exercise of skill and care" a tooth can be filled with gold. And when this view is adopted and acted upon by the profession, there will be but little point for controversy on this subject.

But why is amalgam not as good to fill *fangs* as the *crowns* of teeth? surely it is more exposed in the latter than in the former, and it can be as easily introduced into the fangs as gold.

"When the fangs are well filled, and a few days have elapsed without inflammation, the crown cavity may be as safely filled with amalgam as with gold, so far as periostitis is concerned." The same is true of putty, but that does not prove that it is a good material with which to fill teeth. The liability to produce periostitis is not the great objection to the use of amalgam. There are many, more prominent than this one.

"Never allow any one to say he was deceived by the use of an article in his teeth, which, if he had known the nature of, should not have been put there." This is good advice, and should be adopted by every member of the profession.

Well, I do not know any one who owes the profession more in the way of good advice than Dr. T. It will keep him very busy to cancel what he did in regard to amalgams two years ago.

What is the necessity for keeping amalgam fillings dry while being introduced into the cavity? Amalgam will unite into a solid mass as readily in water or saliva as anywhere else. Gold fillings are kept dry that the portions of gold composing the filling may unite together.

After the amalgam is introduced and united into a mass, and the cavity is full, what is the object in farther packing, consolidating or rubbing? Can a globule of mercury be made to occupy a less space by rubbing or packing, than in its natural condition?

Any tooth that can be filled with amalgam *to be serviceable*, *can* be filled with gold, "by the exercise of skill and care." So then, the plea for the use of amalgam fillings is reduced simply to a matter of economy, of time and money. T.

---

### NEW METHOD OF PLATING.

Gentlemen:—Permit me, through your Journal, to communicate to the profession a discovery that I have made for plating silver, copper or brass with gold foil of any thickness. This may have been used before—if so, I have no knowledge of it, neither can I learn that it has by any dentist.

I will commence on a silver set of teeth, all finished up ready for the mouth; I will suppose that you want to plate them with fine gold foil, with from three to ten thicknesses of No. 6, or No. 8 foil. I have not tried any thicker.

I first free my plate from all grease. The next thing, I take my foil, cut it up in strips from half to one inch square, or in any shape you may wish; after getting my foil all ready I take a small buckskin bag, containing about half an ounce of quicksilver, and gently strike it on the side of the plate you first wish to gild. I then rub it all over the plate with my finger on a piece of buckskin, or canton flannel will do, until it is perfectly silvered over; be careful to have it cover all small indentations. No more mercury is required than to give it the mirror appearance. As soon as that is done, take a pair of tweezers and commence laying on the pieces of foil, until you have the side all covered as perfect as you can; press each piece down with your finger or buckskin as you go along. When you have finished one coat, rub it down hard until it assumes the same appearance as at first, then put on another coat, and keep doing so until you have as many thicknesses as you want, being careful to rub each coat down well, or when heated it will blister. When one side is finished, turn over and serve the other side the same until you have all the foil on you want.

It is better to put mercury on but one side at a time, on account of the mercury acting on the plate while you are plating the other side. When you have finished this process the mercury is to be drawn off by heat. This I do, by having a little oven of sheet-iron, about the size of a pint cup, with a cover, having a false bottom of wire, to prevent the teeth from setting on the bottom of the oven. This small oven I set on legs raised about six inches. In this cup I place my plating, and apply my alcohol lamp underneath. Let this remain from ten to twenty minutes, or more if you wish, for the longer you let the heat remain the richer the color of the gold. Remove the cover and you will see the finest piece of gold plating you ever saw. When cool, if you find any places not covered, apply more mercury on the place, and add more foil and heat again.

When you are satisfied with the amount of foil, and have driven off all the mercury, you can burnish with a blood-stone or steel burnisher, not at first bearing on very hard. Burnish and finish to your own taste. When this plating is properly done, you can melt the plate before the plating will be removed. Soldering on a tooth has no effect on it.

By this process you can make a set of teeth of 14 carat gold, soldered with silver solder, and then plate them *well*, and no dentist can tell them from the finest gold; of course, it will not corrode in the mouth, and will not wear off, neither can you remove it only by the file. It takes me about an hour to plate a full set with four thicknesses of foil.

Yours, N. B. SLAYTON.

Madison, Ind. Dental News Letter.

---

### ABSCESS—DIAGNOSIS OF TOOTHACHE.

There is no more reason why a tooth should ache without sufficient cause, than one of the fingers of the hand should be a cause of pain while in a normal state. When a patient complains of pain in the jaw, temple, or over the whole side of the head and face, and there is no discernable disease of the parts, it is fair to presume, that it is caused by a tooth. Without going into a long description of the various causes of toothache, or pathological conditions of parts, inducing pain, the citation of a few cases may be of use to the young practitioner. A lady of fifty years of age, in general good health, called to see us about pain in her face, by which she had been suffering more or less for four weeks. She had called on a dentist near her residence to save time; she referred the pain to the left inferior bicuspids, both of which were considerably worn; the dentist could not discover in them sufficient cause of suffering, but plugged a small crevice in the second one, about as large as a small pin's head. The pain, however, continued the same; they were examined several times, with no better success; the patient applied to us, stated her case, we examined the teeth and found them as before stated, considerably worn; the first one the most, and in passing a fine pointed probe over the worn surface of it, we found a small opening and the pulp partly dead; we opened the cavity freely and cured the case.

A gentleman called to see us, suffering pain along the inferior maxillary; there was no swelling of the parts; the pain had been endured about one week; he always referred it to the inferior maxillary and the wisdom teeth, second bicuspid and space between them, as the first and second molars had been extracted for some years. The wisdom tooth had a large plug in it, which was removed by his dentist to ascertain whether it was a cause of pain, but it was not found to be, the bicuspid was entirely sound; his dentist not being able to discover any cause of pain, the patient called to see us; we found a very small orifice in the gum, which opened to a remnant of a root which had been left about three years before; this was extracted and the case cured.

A gentleman of nervous temperament was attacked with pain in the head and face, principally the left side; he sent for his medical adviser; he diagnosed it as a case of neuralgia, for which he employed narcotics, chloroform, ether, leeches, purgatives, &c., but all to no purpose. After treating the patient for about one week, during which time he grew much worse, the whole nervous system seemed to become involved; he was at times speechless, he could not swallow his food, he would start off and run across the room involuntary if spoken to, or drop down upon his knees, and perform various gyrations as if crazed. He never complained of his teeth in the least, but his medical adviser called upon us to know if we would examine the case for him, as the patient grew worse under his treatment. We found that a front tooth had been plugged about a month, but had given no pain at the time; we, however, removed the plug and found that it had been plugged over the nerve; the tooth bled and the patient was at once relieved, but did not recover from the nervous excitement for some days. It was the most extraordinary case of suffering we ever witnessed. When pain follows shortly after plugging a tooth, although no pain was experienced at the time, we regard it with suspicion and remove the plug. In this case the nerves of motion, as well as the nerves of general sensibility, were seriously involved.—*News Letter.* J. D. W.

---

### ULCERATIVE STOMATITIS.

The following extract we take from the "Bibliographic Notices" of the Boston Medical and Surgical Journal. We insert it for what it is worth. The idea of promptly curing the disease under all circumstances, with the "specific" remedy suggested is Brandreth like; and the statement that the disease *seems* "to be unknown to some of the most skillful of the dentists" may be true, but it probably seems so to no imagination but that of the writer.

"Finally, we cannot forbear allusion to an important point in relation to the management of the teeth, which would find a most fitting place in a book of this sort. We allude to ulcerative stomatitis, the cause of a vast amount of suffering in the way of toothache and neuralgia of the jaws, and which only requires for its removal the use, for a few days, under the advice of a physician, of chlorate of potash, This is specific, but both the disease and the remedy seem to be unknown to some of the most skillful of the dentists. The following cases, out of some half dozen, have occurred to us in less than a twelvemonth;— A young lady, after ten days' suffering from neuralgia with an inflamed tooth, in whom the alveolar process had been bored with the hope of opening into a supposed abscess at the root, together with a variety of other unpleasant experiments, desired my advice before submitting to extraction. An examination revealed the neck cf the tooth, exposed to its junction with the process, by ulceration of the gum; the tooth itself being elongated, loose, and excessively tender, from the periostitis which always accompanies the affection, but otherwise sound. A few days of the above treatment entirely and effectually relieved the difficulty. When, with a little justifiable malice, she informed the dentist of his mistake, the satisfactory reply was, "Why what a curious name for it; I never heard it before!" In December last, a Canadian gentleman wrote for advice as to "pain in the teeth, jaws and back of the head. My dentist tells me my teeth are good and sound, but that the gums have somewhat receded, and he can apply no remedy. This is consolatory!!" Sure enough: he was relieved in less than a week by chlorate of potash. Last Spring, a patient in fine health, and with a most enviable set of nutcrackers, desired us to look at a tooth which had kept him awake several nights in intense pain. and for which, he had just been informed, the only remedy was extraction, although the tooth was perfectly sound. It was ulcerative stomatitis—nothing else —and a few days sufficed for its permanent relief. We mention these cases in the hope that they may attract attention, and that a few *hints*, well put, will make persons think twice before either *advising* or submitting to the extraction of a sound masticator, the loss of each being such a direct and irremediable injury to the digestive organs."

A Hit at the Doctors.—The Philadelphia Medical and Surgical Journal thinks, that a hard hit at the Medical fraternity is given in Mark's gospel (v. 26), relating to a "certain woman" who "had *suffered many things of many physicians*, and spent all that she had, and was nothing better *but rather grew worse!*" We beg leave to differ with our contemporary in this. The hit in this case is, we think, rather at the patient than the doctors. The fault committed by this woman was one which is still common with those suffering under obstinate chronic diseases, in that she continued to change from one physician to another, instead of sticking to her regular medical adviser. She "*suffered many things from many physicians*"—herein lay the difficulty.

---

Eds. Dental Register:—Please give the following notice an insertion in the December No. of the *Dental Register.*

DENTAL PROGRESS.

The undersigned committee, appointed by the Mississippi Valley Association at its last meeting, to report upon "Dental Progress," would respectfully solicit from members of the profession, any new or improved methods, processes, or appliances relating to the several departments of dental practice that may be considered worthy of note.

It is the desire of the committee to embody in the report, as fully as practicable such principles, methods of practice, and inventions of recent introduction, as shall indicate as briefly and clearly as possible, the actual rate of *progress* in the profession during the current year.

Communications addressed to either member of the committee, should be sent in by the 1st of February, 1858, or sooner.

J. Richardson, Cincinnati. }
J. Taft, " } Committee.
H. R. Smith. " }

### BREATH IMMORTAL.

A lady whose cavernous teeth and crownless stumps emitted an odor annihilating the fragrance of a balmy summer air, had the pleasure of a ride with a gentleman of refined sense, and some cool quiet wit. Proximity in the same buggy and on the same seat brought the gentleman fairly within range of her oracular mouth. The lady announced with a tone of serious confirmation her determination to accomplish a favorite purpose, "*if the Lord did not take her breath.*"—Her gallant escort replied, "You need not be scared—the Lord will not take your breath till you get a better one."

---

### SUBJECTS FOR DISCUSSION.

The business committee appointed at the last meeting of the Mississippi Valley Association, would respectfully submit the following topics for discussion at the meeting in February next:

1. What means will be most efficient in securing a healthy denture?

2. What is the most effectual treatment of exposed nerves, and what circumstances modify the treatment?

3. Filling teeth.

4. What are the indications for the extraction of the deciduous teeth?

5. Irregularity, and its treatment.

6. To what extent may the atmospheric principle be applied to partial sets of teeth?

7. What are the merits and demerits of that method of nserting artificial teeth denominated "*Cheoplasty?*"

J. TAFT,<br>

J. RICHARDSON, } Committee.<br>

J. P. ULLERY,

